# Effects of reproductive condition, roost microclimate, and weather patterns on summer torpor use by a vespertilionid bat

**DOI:** 10.1002/ece3.913

**Published:** 2013-12-20

**Authors:** Joseph S Johnson, Michael J Lacki

**Affiliations:** 1 Department of Biology, Bucknell UniversityLewisburg, 17837, Pennsylvania; 2 Department of Forestry, University of KentuckyLexington, 40546, Kentucky

**Keywords:** Chiroptera, *Corynorhinus rafinesquii*, day-roosts, ecophysiology, heterothermy, microclimates, roosting ecology, thermoregulation

## Abstract

A growing number of mammal species are recognized as heterothermic, capable of maintaining a high-core body temperature or entering a state of metabolic suppression known as torpor. Small mammals can achieve large energetic savings when torpid, but they are also subject to ecological costs. Studying torpor use in an ecological and physiological context can help elucidate relative costs and benefits of torpor to different groups within a population. We measured skin temperatures of 46 adult Rafinesque's big-eared bats (*Corynorhinus rafinesquii*) to evaluate thermoregulatory strategies of a heterothermic small mammal during the reproductive season. We compared daily average and minimum skin temperatures as well as the frequency, duration, and depth of torpor bouts of sex and reproductive classes of bats inhabiting day-roosts with different thermal characteristics. We evaluated roosts with microclimates colder (caves) and warmer (buildings) than ambient air temperatures, as well as roosts with intermediate conditions (trees and rock crevices). Using Akaike's information criterion (AIC), we found that different statistical models best predicted various characteristics of torpor bouts. While the type of day-roost best predicted the average number of torpor bouts that bats used each day, current weather variables best predicted daily average and minimum skin temperatures of bats, and reproductive condition best predicted average torpor bout depth and the average amount of time spent torpid each day by bats. Finding that different models best explain varying aspects of heterothermy illustrates the importance of torpor to both reproductive and nonreproductive small mammals and emphasizes the multifaceted nature of heterothermy and the need to collect data on numerous heterothermic response variables within an ecophysiological context.

## Introduction

Heterothermic small mammals are notable for their ability to enter a state of metabolic suppression known as torpor (Geiser 2004; Storey et al. 2010; Geiser and Brigham 2012). Torpor is often recognized as hibernation, a winter survival strategy essential to many temperate zone species. It is becoming increasingly apparent, however, that torpor is used throughout the year for numerous physiological purposes, revising our understanding of the costs and benefits associated with torpor outside of hibernation (Geiser and Brigham 2012; Dzal and Brigham 2013). It is also becoming increasingly apparent that the number of heterothermic mammals is quite large, and not restricted to species inhabiting the temperate zones (Bartels et al. 1998; Geiser 2004; Dausmann et al. 2005; Bondarenco et al. 2013). Torpor is undeniably a powerful adaptation, an adaptation frequently used in response to internal stressors such as energetic demands, as well as environmental stressors such as weather (McLean and Speakman 1999; Willis et al. 2006; Schmid and Speakman 2009; Dzal and Brigham 2013).

Torpor use by reproductive females (Willis et al. 2006; Dzal and Brigham 2013) is an emerging example of how we must revise our understanding of the costs and benefits of torpor. The physiological cost of reproduction can be high for small mammals, especially during lactation, when caloric demands are greatest (Speakman 2008). Despite these energetic costs, torpor has traditionally been viewed as an energy conservation strategy that reproductive females should avoid because torpor is associated with a reduction in nearly all physiological processes (Storey et al. 2010; Franco et al. 2013), diverting energy away from fetal development and milk production (Racey and Swift 1981; Wilde et al. 1999). Recent studies have challenged this view of torpor, contending that delaying parturition may be beneficial for both mothers and young and that torpor may be the only means some small mammals have to meet the energetic cost of lactation (McLean and Speakman 1999; Willis et al. 2006; Dzal and Brigham 2013). Because the ecological and physiological costs and benefits associated with torpor are expected to be different for individuals not burdened with the demands of reproduction, such as males and nonreproductive females, nonreproductive individuals are sometimes documented using different thermoregulatory strategies during the reproductive season (Hamilton and Barclay 1984; Cryan and Wolf 2003; Lausen and Barclay 2003; Rambaldini and Brigham 2008; Johnson and Lacki 2013). These thermoregulatory strategies are characterized by heterothermic response variables, which include minimum and average body temperature (*T*_b_), as well as the frequency, depth (extent of reduction in *T*_b_), and duration of torpor. Heterothermy in small mammals is more complex than the frequency of torpor use, but instead has numerous facets, each of which can help achieve energetic savings in different ways. For example, a small mammal using relatively deep torpor bouts for a relatively short period of time will experience different costs and benefits compared with an individual of the same species using shallower, but longer torpor bouts (Speakman and Thomas 2003; Storey et al. 2010). This phenomenon can be explained by an understanding of how torpid mammals save energy.

As a result of metabolic suppression during torpor, *T*_b_ typically decreases until it approaches the temperature of the surrounding air, reducing the energetic cost of thermoregulation and other biological processes (Speakman and Thomas 2003; Storey et al. 2010). Heterothermic mammals can experience dramatic decreases in *T*_b_ during torpor at low temperatures, but some species are able to use torpor while resting near the lower limit of their zone of thermal neutrality (Heldmaier and Elvert 2004). Total energetic savings from torpor are maximized at low *T*_b_s due to temperature-dependent reductions in biochemical processes (Storey et al. 2010), but initial drops from normothermic *T*_b_ result in the largest incremental gains in energy savings (Studier 1981; Webb et al. 1993). Several species have been documented using relatively shallow torpor during the summer, presumably to save energy while minimizing ecological costs (Rambaldini and Brigham 2008; Johnson and Lacki 2013). Although torpor can provide significant energetic savings, the benefit of these savings is poorly understood in respect to the costs of torpor, particularly impacts on reproduction, vulnerability to predation, and cost required to raise *T*_b_ back to a normothermic temperature.

Understanding the benefits of using torpor on any given day requires an assessment of environmental temperatures inside and outside the shelter used by a small mammal. Small mammals often choose a single daily shelter from a suite of available shelters, each offering different thermal environments and, therefore, different pathways for energetic savings. Shelters with microclimates within the thermal neutral zone will minimize energy required to maintain a high core *T*_b_, while shelters with relatively cold microclimates hold greater potential for energetic savings through torpor. Thus, we expect torpor use not only to vary among individuals of different sex and reproductive conditions within a population, but also among individuals inhabiting shelters with microclimates better suited to different thermoregulatory strategies.

Other intrinsic and extrinsic factors are also known to influence heterothermic behaviors. Research has shown that torpor use in small mammals is inversely related to food consumption (Audet and Thomas 1997; Bozinovic et al. 2007; Nespolo et al. 2010) and estimates of body condition (Rambaldini and Brigham 2008), suggesting torpor may be used in response to poor foraging success. Because ability to successfully forage may be related to weather events such as periods of extensive rain or cold, we expect torpor use to vary in response to local weather. Thus, torpor is an important adaptation used by small mammals in response to numerous external and internal stressors, and the costs and benefits of torpor are expected to vary within and among species with different physiological conditions in different ecological contexts.

The purpose of our research was to study summer torpor in such an ecological and physiological context and to evaluate four competing *a priori* hypotheses explaining differences in thermoregulatory strategies used by an insectivorous heterothermic mammal, Rafinesque's big-eared bat (*Corynorhinus rafinesquii*). We evaluated reproductive, microclimate, and weather-driven hypotheses for explaining differences in torpor frequency, depth, and duration, among different sex and reproductive classes of bats roosting in diurnal shelters representing a spectrum of thermal habitats. We predicted that torpor variables would not be consistently explained by a single hypothesis, but that torpor strategies would differ under various physiological (reproductive condition) and ecological (microclimate and weather) conditions.

## Materials and Methods

### Study area

**Figure 1:**
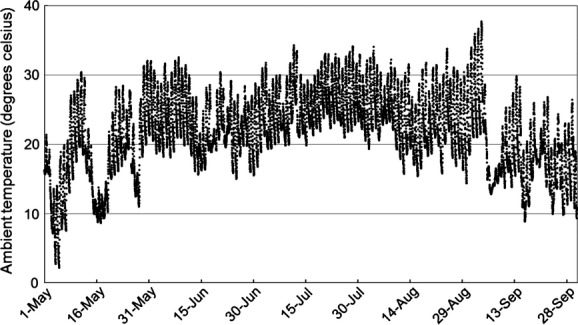
Ambient outdoor temperatures recorded at Mammoth Cave National Park, Kentucky, USA, May–September, 2011.

Field work was conducted during the summer maternity season (May–September) from 2009 to 2011, at Mammoth Cave National Park, located in central Kentucky, USA (37.2072°N, 86.1319°W). The Park is predominantly forested, consisting primarily of oak-hickory (*Quercus*-*Carya* spp.) and western mixed mesophytic forests (Braun 1950). Erosion of the limestone and sandstone bedrock by numerous small and large drainages has created a topographically diverse landscape containing hundreds of small caves, as well as the longest known cave system in the world. Small caves and rock shelters located in sandstone cliff lines, hollow trees, and abandoned human structures serve as roosts for Rafinesque's big-eared bats within the Park. These roosts represent a spectrum of thermal habitats ranging from roosts with stable temperatures markedly colder than outside ambient air temperature (*T*_outside_) (caves), to roosts with variable temperatures (buildings, trees, and shelters), including roosts markedly warmer than *T*_outside_ (building attics). *T*_outside_ during the middle of the study period, June-August, typically ranged between 20 and 35°C, with colder, more variable temperatures during May and September (Fig. 1).

### Data collection

Bats were captured in polyester mist nets (Avinet, Inc., Dryden, NY) placed over roads, ponds, and outside entrances to known roosts. All bats were classified as adult or juvenile by examining epiphyseal–diaphyseal fusions of long bones in the wing (Brunet-Rossinni & Wilkinson 2009). Female reproductive condition was determined as pregnant, lactating, or postlactating based on the presence of a fetus or teat condition (Racey 2009). Females with no sign of a fetus or lactation were determined to be nonreproductive. When possible, we affixed a 0.42 g temperature-sensitive, or nontemperature-sensitive, radio transmitter (model LB-2N-T and LB-2N, Holohil Systems, Ltd., Carp, Ontario) between the shoulder blades of adult males and females using a surgical adhesive (Torbot, Cranston, RI; Perma-Type, Plainville, CT).

Bats were tracked to day-roosts every day until radio transmitters fell off or until we believed the battery had expired. An account of the day-roosts used by each bat, including the type of roost (cave, man-made structure, rock shelter, or tree cavity) used, was recorded for each day a radio transmitter remained attached and functional. Roost-switching frequencies were quantified for each bat we could consistently locate (i.e., those we did not fail to locate for >1 consecutive day) by dividing the number of days a bat was successfully tracked to a roost by the number of days that a bat switched its roost.

Three datalogging receivers (model R4500S, Advanced Telemetry Systems, Inc., Isanti, MN) were deployed at various locations throughout the Park and programmed to record the pulse rates of temperature-sensitive radio transmitters at 5-min intervals. Receivers were left in the field throughout the entire study period. Receivers were checked each morning and moved to a new location when necessary to maximize the detection of radio signals. Temperature-sensitive radio transmitters were individually calibrated by the manufacturer, allowing us to create a unique polynomial equation for each transmitter to convert recorded pulse rates into skin temperatures (*T*_sk_) for each radio-tagged bat.

Microclimate dataloggers (model U23-001, Onset Computer Corporation, Bourne, MA) were placed inside several roosts to measure roost temperatures (*T*_cave_, *T*_tree_, *T*_shelter_, *T*_attic_, or in general, *T*_roost_). Within tree hollows, we attempted to consistently place dataloggers at a height of 4 m above the ground, approximately 1 m below the height where we commonly observed bats roosting. Within caves, rock shelters, and man-made structures, we mounted dataloggers in the general location where bats were observed roosting. Because bats would often roost in groups of >100 in building attics and caves, care was taken to place dataloggers in a location close to where bats roosted, while avoiding direct contact with the clusters of bats. Dataloggers were programmed to record microclimate measurements at 15-min intervals and were checked at 6-month intervals for maintenance or removal.

Three dataloggers were placed inside solar radiation shields to record *T*_outside_ throughout the study area. *T*_outside_ was recorded at the same 15-min intervals as *T*_roost_ to allow for comparison of roost conditions to local weather. For each 15-min interval, *T*_roost_ – *T*_outside_ was calculated using the *T*_outside_ datalogger located closest to each roost. Each *T*_roost_ and *T*_outside_ measurement was classified as a daytime or nighttime measurement based upon local sunrise and sunset times. For each full day of microclimate sampling within a roost, we calculated average daytime and nighttime *T*_roost_, daytime and nighttime variance in *T*_roost_, average daytime and nighttime *T*_roost_ – *T*_outside_, and daytime and nighttime variance in *T*_roost_ – *T*_outside_. Data for daily precipitation were obtained from a weather station proximally located (26 km) to the study site (Weather Underground Inc. 2013).

### Data analysis

A torpor onset (*T*_onset_) cutoff value was used to determine whether each *T*_sk_ reading represented a torpid or normothermic temperature. This cutoff was determined using the equation in Willis (2007) using model parameters minus 1 SE. Although the equation presented in Willis (2007) was designed for use with (*T*_b_) data, the equation has been applied to *T*_sk_ data given the tight correlation between *T*_sk_ and *T*_b_ in small mammals (Barclay et al. 1996; Dzal and Brigham 2013; Johnson and Lacki 2013). Because this equation requires simultaneous measures of *T*_sk_ and *T*_roost_, *T*_onset_ was calculated only on days that bats occupied roosts with microclimate dataloggers. Because *T*_onset_ varied minimally (mean = 32.1°C, range = 31.5−32.4°C), a *T*_onset_ value of 32°C was applied to all *T*_sk_ recordings for analysis. Bats were considered torpid when *T*_sk_ fell below 32°C for two consecutive data points (i.e., for >10 min).

The *T*_onset_ threshold was used to determine the daily number of torpor bouts used, total time spent torpid, average depth of torpor bouts, average *T*_sk_, and minimum *T*_sk_ for each day of data collection for each bat. For bats with only a single torpor bout on any given day, average depth of a torpor bout was calculated by averaging *T*_sk_ – *T*_onset_ for each *T*_sk_ reading below *T*_onset_. For bats with multiple torpor bouts on any given day, average depth of each torpor bout was calculated and then averaged among bouts. Four linear mixed models (LMMs), each representing a unique *a priori* hypothesis, were created to explain the variation in each dependent variable. Each dependent variable was assessed individually, and the same four models were used to evaluate each variable. The most parsimonious model for each dependent variable was determined using Akaike's information criterion adjusted for small sample sizes (AIC_c_), ranking models using Akaike differences (Δ_*i*_) and weights (*ω*_*i*_). The model intercept and error terms were used as parameters in calculation of AIC_c_ (Burnham and Anderson 2002). Analyses of average torpor bout depth were based on the dataset limited to bat-days when torpor was used. LMMs were used because they allow for inclusion of individual radio-tagged bats as random variables, preventing pseudoreplication, while incorporating day-to-day variability in individual torpor behaviors. This random variable was handled differently in each of the four competing models.

The *reproductive condition hypothesis* predicts that differences in torpor use are best explained by the sex and reproductive condition of the individual. This model uses sex and reproductive condition as the independent variable, nesting individual bat identity as a random variable. The *roost microclimate hypothesis* predicts that differences are best explained by *T*_roost_. This model uses roost types, caves, man-made structures, trees, and sandstone rock shelters, as the independent variable and nested individuals as random variables. We compared measures of *T*_roost_ and *T*_roost_ – *T*_outside_ among roost types using a LMM for each variable, with individual roosts as a random variable, to test our hypothesis that microclimates differed among the four roost types. We used a significance threshold of 0.05 and Fisher's least significant difference to compare mean values from significant tests. Three abandoned buildings had multiple levels, including basements, ground levels, and attics used by roosting bats. Because bats typically roosted in attics this analysis was limited to *T*_attic_. The *current weather hypothesis* predicts that differences in torpor are best explained by weather variables that directly affect roost selection and conditions inside the roost: *T*_outside_ at sunrise, daily minimum *T*_outside_, and total daily precipitation. Lastly, the *past weather hypothesis* predicts that differences in torpor are best predicted by weather variables that affect foraging success: *T*_outside_ at sunset on the previous day, daily minimum *T*_outside_ during the previous night, and total daily precipitation from the previous day. Both weather models used weather variables as independent variables and individual bats as a random variable.

## Results

Fifty-nine adult Rafinesque's big-eared bats were radio-tagged, including 12 males, 10 pregnant, 13 lactating, 18 postlactating, and 6 nonreproductive females, between 2009 and 2011. Radio-tagged bats weighed 10.5 g ± 0.2 (SE). Bats were successfully located on 705 of 739 (95%) bat-days (1 bat day = 1 day of data from 1 bat). We located bats roosting in 10 caves, 11 rock shelters, 11 tree cavities, and 10 man-made roosts. Bats also roosted on the outside of five live trees. Bats (*n *=* *57) switched roosts an average of every 6.0 ± 0.5 day. One male and one postlactating female could not be consistently located and were excluded from this summary of roost switching.

*T*_sk_ data were collected on 413 bat-days from 46 bats, including 7 males, 10 pregnant, 13 lactating, 11 postlactating, and 5 nonreproductive females (Table 1). Torpor use was documented on 215 days (52%). Torpor was used on ≥1 day by 41 radio-tagged bats (89%), including bats from all sex and reproductive classes. Morning torpor bouts, beginning between sunset and 0800 hours, were documented on 106 days (49%). Evening torpor bouts, beginning between 1700 hours and sunset, were documented on 61 days (28%). Both morning and evening torpor were documented on 16 days (7%). Nighttime torpor bouts, beginning and ending between sunset and sunrise, were documented on 26 days (12%).

**Table 1:** Summary of summer torpor use among sex and reproductive classes of Rafinesque's big-eared bats roosting in different roosting structures at Mammoth Cave National Park, Kentucky, USA, 2009–2011. Sample sizes for each sex and reproductive class are reported in parentheses.

	Bouts per day	Avg. time torpid (h/day)	Avg. bout depth (°C)	Minimum *T*_sk_ (°C)	Average *T*_sk_ (°C)
Sex and reproductive class					
Males (7)	0.5 ± 0.7	1.3 ± 2.6	−3.4 ± 3.7	31.5 ± 3.8	34.2 ± 2.5
Pregnant (10)	0.9 ± 1.3	0.9 ± 2.0	−1.7 ± 2.7	31.0 ± 3.2	34.4 ± 2.0
Lactating (13)	1.1 ± 1.4	1.6 ± 3.4	−1.3 ± 1.2	31.3 ± 2.9	34.9 ± 2.0
Postlactating (11)	1.2 ± 1.3	2.6 ± 4.1	−2.7 ± 2.9	29.7 ± 4.1	33.6 ± 3.0
Nonreproductive (5)	2.0 ± 1.5	4.5 ± 4.7	−2.1 ± 2.5	28.0 ± 4.4	32.4 ± 3.2
Roost types					
Caves	2.0 ± 1.5	3.9 ± 4.8	−2.4 ± 2.9	28.6 ± 3.7	32.6 ± 3.2
Rock shelters	0.8 ± 1.2	1.8 ± 3.0	−3.7 ± 3.4	31.0 ± 4.2	34.4 ± 3.1
Tree cavities	0.6 ± 1.0	2.2 ± 5.0	–1	30.2 ± 5.1	34.0 ± 3.9
Buildings	0.8 ± 1.2	1.3 ± 2.7	−1.8 ± 2.3	31.0 ± 3.5	34.5 ± 2.0

1 Data for skin temperatures collected from bats roosting in trees were not used in model selection of average torpor depth due to insufficient sample size (*n *=* *4 days where torpor use).

**Figure 2:**
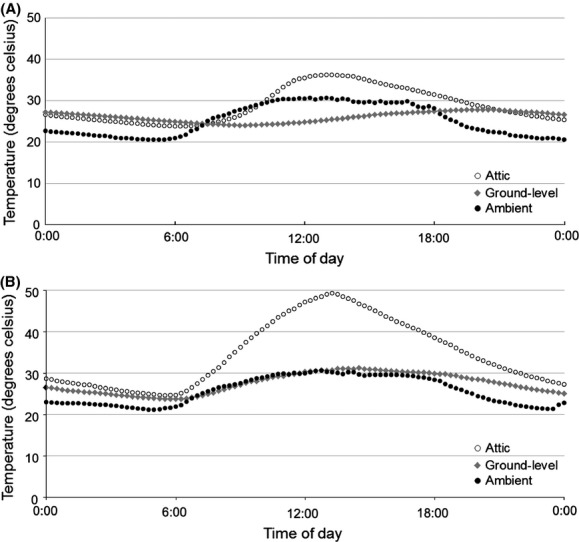
Temperatures inside building roosts used by Rafinesque's big-eared bats, highlighting different microclimates available within buildings at Mammoth Cave National Park, Kentucky, USA.

A total of 3630 roost days (1 roost day = 1 day of data from 1 roost) of microclimate data were collected from 26 roosts, including 6 caves, 7 man-made structures, 5 trees, and 8 sandstone rock shelters. Man-made structures included 6 buildings and 1 aboveground concrete cistern. An additional 444 roost days of data were collected from basements and ground floor levels of buildings, but were not included in analyses because bats typically roosted in attics (Fig. 2). Daytime average *T*_roost_ (*F*_3,22_ = 15.5, *P *<* *0.001), nighttime average *T*_roost_ (*F*_3,22_ = 9.1, *P *<* *0.001), daytime variance in *T*_roost_ (*F*_3,22_ = 8.0, *P* = 0.001), nighttime variance in *T*_roost_ (*F*_3,22_ = 11.5, *P *<* *0.001), daytime average *T*_roost_ – *T*_outside_ (*F*_3,22_ = 17.7, *P *<* *0.001), nighttime average *T*_roost_ – *T*_outside_ (*F*_3,22_ = 9.8, *P *<* *0.001), and nighttime variance in *T*_roost_ – *T*_outside_ (*F*_3,22_ = 3.6, *P *=* *0.03) differed among roost types (Table 2). Daytime variance in *T*_roost_ – *T*_outside_ did not differ among roost types (*F*_3,22_ = 0.31, *P *=* *0.82).

**Table 2:** Summary of roost temperatures (*T*_roost_) and differences between outdoor air (*T*_outside_) and roost temperatures (*T*_roost_ – *T*_outside_) among roosting structures used by Rafinesque's big-eared bats during the summer (May–September) in Kentucky, USA, 2009–2011. Sample sizes are reported in parentheses.

Variable	Caves (6)	Rock shelters (8)	Tree cavities (5)	Buildings (7)
Daytime average *T*_roost_	17.4 ± 1.2^a^	19. 6 ± 1.0^a,b^	22.2 ± 1.3^b^	27.4 ± 1.1^c^
Daytime *T*_roost_ variance	0.09 ± 3.5^a^	0.60 ± 3.0^a^	1.9 ± 3.9^a^	19.4 ± 3.3^b^
Daytime average *T*_roost_ – *T*_outside_	−6.6 ± 1.0^a^	−4.4 ± 0.8^a,b^	−2.0 ± 1.1^b^	2.8 ± 0.9^c^
Daytime *T*_roost_ – *T*_outside_ variance	8.9 ± 1.7	7.4 ± 1.5	6.6 ± 1.9	7.7 ± 1.5
Nighttime average *T*_roost_	17.3 ± 1.1^a^	19.1 ± 1.0^a,b^	21.6 ± 1.2^b,c^	24.4 ± 1.0^c^
Nighttime *T*_roost_ variance	0.08 ± 0.7^a^	0.36 ± 0.6^a^	1.5 ± 0.8^a^	4.5 ± 0.7^b^
Nighttime average *T*_roost_ – *T*_outside_	−2.7 ± 0.9^a^	−0.65 ± 0.8^a,b^	1.9 ± 1.0^b,c^	3.6 ± 0.9^c^
Nighttime *T*_roost_ – *T*_outside_ variance	2.8 ± 0.4^a^	1.8 ± 0.3^a,b^	1.1 ± 0.4^b^	1.7 ± 0.3^b^

For each variable, roosting structures not sharing common superscript letters for a variable were significantly different at *P *<* *0.05.

Heterothermic response variables were best explained by different models. The reproductive condition hypothesis received the strongest support for explaining two variables: the average time spent torpid each day and the average depth of torpor bouts. Although no other models received strong support (Δ_*i*_ < 2) for either variable, several models received some support (Δ_*i*_ < 4; Table 3). The roost microclimate hypothesis received the greatest support for explaining the average number of torpor bouts used each day, with no other hypotheses receiving strong support (Table 3). We removed data from bats roosting in trees from the analysis of average torpor bout depth due to low sample size (*n *=* *4 days where bats used torpor). The current weather hypothesis received the greatest support for explaining average and minimum *T*_sk_, again, with no other models receiving strong support (Table 3).

**Table 3:** Akaike's information criterion (AIC_c_) scores, differences (Δ_*i*_), weights (*w*_*i*_), and number of parameters (K) from linear mixed models explaining five different heterothermy response variables collected using radio telemetry on Rafinesque's big-eared bats in Kentucky, USA, 2009–2011.

Variable and hypothesis	K	AICc	Δ_*i*_	*w*_*i*_
Number of torpor bouts				
Reproductive condition	4	1377	29	<0.001
Roost microclimate^1^	4	1348	0	0.98
Current weather	6	1357	8.2	0.16
Past weather	6	1361	13	0.001
Average time spent torpid				
Reproductive condition^1^	4	2125	0	0.68
Roost microclimate	4	2129	3.9	0.10
Current weather	6	2128	3.2	0.14
Past weather	6	2129	4.3	0.08
Average depth of bouts				
Reproductive condition^1^	4	958	0	0.65
Roost microclimate	4	962	4.2	0.08
Current weather	6	961	3.5	0.12
Past weather	6	960	2.9	0.15
Average T_sk_				
Reproductive condition	4	1872	23	<0.001
Roost microclimate	4	1866	16	<0.001
Current weather^1^	6	1849	0	0.98
Past weather	6	1857	7.5	0.02
Minimum T_sk_				
Reproductive condition	4	2144	60	<0.001
Roost microclimate	4	2144	60	<0.001
Current weather^1^	6	2084	0	0.99
Past weather	6	2098	14	0.001

1 Denotes most parsimonious model.

## Discussion

The use of torpor to conserve energy is well documented among bats (Speakman and Thomas 2003), with other uses of torpor receiving an increasing amount of attention (Geiser and Brigham 2012). Our results add to the growing body of research demonstrating the importance of torpor to reproductive females during the summer (Geiser et al. 2005; Turbill and Geiser 2006; Dzal and Brigham 2013). Unlike other studies, however, we provide an assessment of thermoregulatory strategies among males, pregnant, lactating, postlactating, and nonreproductive females in an ecophysiological context to identify how reproductive condition and environmental variables contribute to the patterns observed in summer heterothermy. Interestingly, measures of heterothermy in Rafinesque's big-eared bats most influenced by sex and reproductive status were not the same measures that were most affected by environmental stresses. Thus, our study illustrates that understanding heterothermy requires examination of a suite of variables representing different components of thermoregulatory strategies.

**Figure 3:**
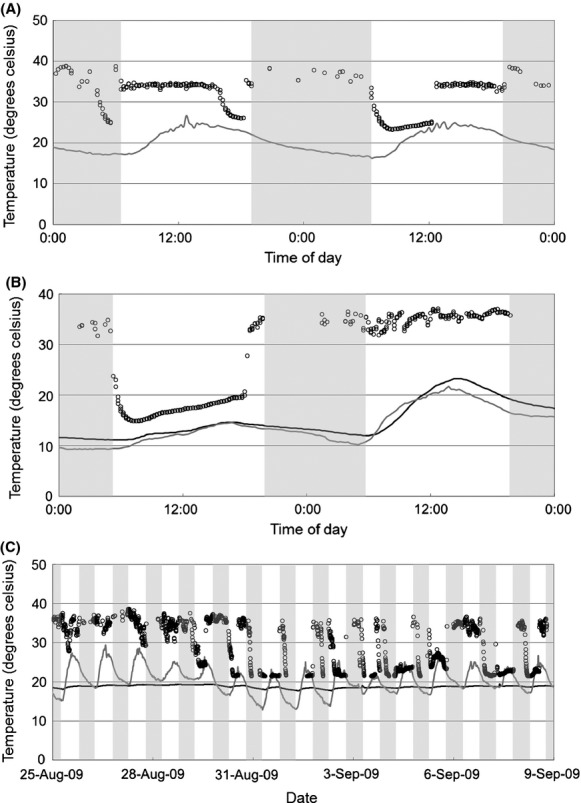
Skin temperature profiles (open circles) of male (A), pregnant female (B), and postlactating female (C) Rafinesque's big-eared bats, alongside outdoor air (gray line) and roost (black line, when available) temperatures recorded at Mammoth Cave National Park, Kentucky, USA. Areas shaded in gray represent time between sunset and sunrise.

The reproductive condition hypothesis best explained the amount of time bats spent torpid and the average depth of their torpor bouts. Reproductive females used shorter, shallower torpor bouts than non-or postreproductive females. Pregnant females spent the least amount of time torpid each day, and pregnant and lactating females used shallower (higher torpid *T*_sk_) torpor bouts compared with postlactating and nonreproductive females. These findings are consistent with the long-held belief that torpor use by reproductive females comes with physiological costs, including reductions in fetal development (Racey and Swift 1981; Speakman 2008) and milk synthesis (Wilde et al. 1999). Similar to other recent research, however, we frequently observed torpor use during reproduction despite these apparent costs (Turbill and Geiser 2006; Dzal and Brigham 2013; Johnson and Lacki 2013). Among reproductive females, we found lactating females using torpor for the greatest amount of time per day, possibly helping to compensate for increased energy expenditures during lactation (McLean and Speakman 1999). It is important to note, however, that we began collecting *T*_sk_ data late in the gestation period. Colder, more variable environmental conditions were more frequent earlier in gestation and may have favored increased use of deep, prolonged torpor bouts by pregnant females (Willis et al. 2006). This is supported by occasional observations of *T*_sk_ as low as 15°C in pregnant females during mid-May (Fig. 3).

Longer, deeper torpor bouts were more common in nonreproductive females, especially postlactating females during September (Fig. 3). These females used torpor bouts lasting several hours during both day and night, a phenomenon we also observed among nonreproductive females during this time. We hypothesize that torpor is especially important to Rafinesque's big-eared bats as a mechanism to gain weight in preparation for hibernation. Although conditions within the study area can be relatively warm throughout September, ranging 10–30°C, we hypothesize that availability of moths, the preferred prey of big-eared bats (Lacki and Dodd 2011), declines significantly after August, limiting foraging capabilities during this critical time. Speakman and Rowland (1999) found that captive brown long-eared bats (*Plecotus auritus*), given a choice between warm and cold roosts, selected colder roosts during the prehibernation period. Speakman and Rowland (1999) observed bats using torpor extensively during this time and found that bats achieved a positive energy balance despite reductions in digestive efficiency resulting from torpor. Our results from free-ranging Rafinesque's big-eared bats mirror these findings. Bats in our study likely gather seasonal cues to prepare for hibernation from changing photoperiod, temperatures, and insect availability. We postulate that these bats increase their torpor use during late summer and throughout the fall as a mechanism to gain fat reserves needed to survive the winter.

The roost microclimate hypothesis was the best model describing the number and depth of torpor bouts used each day. Bats roosting in caves used torpor at approximately twice the frequency of bats roosting in other roost types. This was expected given the cold, stable temperatures in cave roosts; however, it was surprising that the roost microclimate model did not gain more support in explaining other heterothermic response variables. We postulate that social thermoregulation had a strong influence on the heterothermic responses of bats roosting in caves, resulting in low support for the roost microclimate model. The presence of a bat colony on roost temperatures is significant, decreasing the amount of energy required to remain normothermic in roosts with temperatures below the thermal neutral zone (Willis and Brigham 2007). The influence of social behavior on thermoregulation was also demonstrated by Franco et al. (2012), who observed higher *T*_b_s in *Dromiciops gliroides* huddling in groups compared with single individuals at 20°C because solitary individuals quickly entered torpor. In our study, irregular evening emergence counts showed that caves were typically inhabited by colonies of 50–100 bats, likely providing Rafinesque's big-eared bats with opportunities for clustering and decreased costs of remaining normothermic. We propose that caves are valuable and unique roosts in our study area because they provide this opportunity for multiple thermoregulatory strategies. By clustering, bats in caves can defend normothermic temperatures at minimum cost, yet can access colder temperatures conducive to large energetic savings by simply moving within the roost. This may explain why bats used torpor at twice the frequency as bats in roosts with temperatures more affected by fluctuations in *T*_outside_.

Finally, the current weather hypothesis best explained average and minimum *T*_sk_. While average torpor bout duration takes both the amount of time spent torpid and reduction in *T*_sk_ into account, minimum *T*_sk_ represents daily maximum torpor depth. Our results show that daily weather conditions, including *T*_outside_ at sunrise, average daily *T*_outside_, and total daily precipitation, best determine minimum *T*_sk_. These results are intuitive alongside our finding that many bats used torpor after returning to their roosts during the early morning hours, when *T*_outside_ is near its daily minimum. Unlike minimum *T*_sk_, average *T*_sk_ relates information about torpid and normothermic temperatures. Johnson and Lacki (2013) found that nonreproductive female big-eared bats roosting in tree cavities had the lowest average *T*_sk_, just above the torpor onset threshold, while pregnant females maintained the highest average *T*_sk_. The present study of bats roosting in a variety of thermal habitats found average *T*_sk_ was best explained by daily weather.

Our study presents a view of torpor in Rafinesque's big-eared bat where different characteristics of torpor behavior are best explained by differing physiological or environmental conditions. In this view, only differences in average torpor depth and amount of time spent torpid are best explained by reproductive condition, whereas roost temperatures and daily weather variables explain frequency of torpor use and minimum and average *T*_sk_. These results not only provide insight into the torpor behaviors of this species, but also provide an understanding of how to quantify heterothermy in other small mammals, which has been a topic of considerable debate (Willis 2007; Boyles et al. 2011a,b2011b; Brigham et al. 2011). Heterothermy in small mammals is driven by both internal metabolism and fluctuations in environmental temperatures. Our study illustrates that attempting to quantify heterothermy using a single variable (Boyles et al. 2011a) may be inadequate because torpor has several characteristics that are influenced by different internal and external stimuli. The variables measured in this study, frequency of torpor use, daily time spent torpid, average duration of torpor bouts, and minimum and average *T*_sk_, and the models that most parsimoniously explain them, illustrate how quantifying heterothermy using a single variable may mask important differences in the ways sex and reproductive classes of small mammals thermoregulate.

## References

[b1] Audet D, Thomas DW (1997). Facultative hypothermia as a thermoregulatory strategy in the phyllostomid bats, *Carollia perspicillata* and *Sturnira lilium*. J. Comp. Physiol. B.

[b2] Barclay RMR, Kalcounis MC, Crampton LH, Stefan C, Vonhof MJ, Wilkinson L (1996). Can external radiotransmitters be used to assess body temperature and torpor in bats?. J. Mammal.

[b3] Bartels W, Law BS, Geiser F (1998). Daily torpor and energetics in a tropical mammal, the northern blossom-bat *Macroglossus minimus* (Megachiroptera). J. Comp. Physiol. B.

[b4] Bondarenco A, Körtner G, Geiser F (2013). Some like it cold: summer torpor by freetail bats in the Australian arid zone. J. Comp. Physiol. B.

[b5] Boyles JG, Smit B, McKechnie AE (2011a). A new comparative metric for estimating heterothermy in endotherms. Physiol. Biochem. Zool.

[b6] Boyles JG, Smit B, McKechnie AE (2011b). Does use of the torpor cut-off method to analyze variation in body temperature cause more problems than it solves?. J. Therm. Biol.

[b7] Bozinovic F, Muñoz JL, Naya DE, Cruz-Neto AP (2007). Adjusting energy expenditures to energy supply: food availability regulates torpor use and organ size in the Chilean mouse-opossum *Thylamys elegans*. J. Comp. Physiol. B.

[b8] Braun EL (1950). Deciduous forests of eastern North America.

[b9] Brigham RM, Willis CKR, Geiser F, Mzilikazi N (2011). Baby in the bathwater: should we abandon the use of body temperature thresholds to quantify expression of torpor?. J. Therm. Biol.

[b100] Brunet-Rossinni AK, Wilkinson GS, Kunz TH, Parsons S (2009). Methods for age estimation and the study of senescence in bats. Ecological and behavioral methods for the study of bats.

[b10] Burnham KP, Anderson DR (2002). Model selection and multi-model inference: a practical information-theoretic approach.

[b11] Cryan PM, Wolf BO (2003). Sex differences in the thermoregulation and evaporative water loss of a heterothermic bat, *Lasiurus cinereus*, during its spring migration. J. Exp. Biol.

[b12] Dausmann KH, Glos J, Ganzhorn JU, Heldmaier G (2005). Hibernation in the tropics: lessons from a primate. J. Comp. Physiol. B.

[b13] Dzal YA, Brigham RM (2013). The tradeoff between torpor use and reproduction in little brown bats (*Myotis lucifugus*. J. Comp. Physiol. B.

[b14] Franco M, Contreras C, Cortés P, Chappell MA, Soto-Gamboa M, Nespolo RF (2012). Aerobic power, huddling and the efficiency of torpor in the South American marsupial, *Dromiciops gliroides*. Biol. Open.

[b15] Franco M, Contreras C, Nespolo RF (2013). Profound changes in blood parameters during torpor in a South American marsupial. Comp. Biochem. Physiol. A: Mol. Integr. Physiol.

[b16] Geiser F (2004). Metabolic rate and body temperature reduction during hibernation and daily torpor. Annu. Rev. Physiol.

[b17] Geiser F, Brigham RM, Ruf T, Bieber C, Arnold W, Millesi E (2012). The other functions of torpor. Living in a seasonal world.

[b18] Geiser F, McAllan BM, Brigham RM (2005). Daily torpor in a pregnant dunnart (*Sminthopsis macroura* Dasyuridae: Marsupalia). Mamm. Biol.

[b19] Hamilton IM, Barclay RMR (1984). Patterns of daily torpor and day-roost selection by male and female big brown bats (*Eptesicus fuscus*. Can. J. Zool.

[b20] Heldmaier G, Elvert R, Barnes BM, Carey HV (2004). How to enter torpor: thermodynamic and physiological mechanisms of metabolic depression. Life in the cold: evolution, mechanisms, adaptation, and application.

[b21] Johnson JS, Lacki MJ (2013). Summer heterothermy in Rafinesque's big-eared bats (Corynorhinus rafinesquii) roosting in tree cavities in bottomland hardwood forests. J. Comp. Physiol. B.

[b22] Lacki MJ, Dodd LE, Loeb SC, Lacki MJ, Miller DA (2011). Diet and foraging behavior of *Corynorhinus* bats in eastern North America. Conservation and Management of Big-eared Bats: A Symposium.

[b23] Lausen CL, Barclay RMR (2003). Thermoregulation and roost selection by reproductive female big brown bats (*Eptesicus fuscus*) roosting in rock crevices. J. Zool.

[b24] McLean JA, Speakman JR (1999). Energy budgets of lactating and nonreproductive brown long-eared bats (*Plecotus auritus*) suggest females use compensation in lactation. Funct. Ecol.

[b25] Nespolo RF, Verdugo C, Cortés PA, Bacigalupe LD (2010). Bioenergetics of torpor in the Microbiotherid marsupial, Monito del Monte (*Dromiciops gliroides*): the role of temperature and food availability. J. Comp. Physiol. B.

[b26] Racey PA, Kunz TH, Parsons S (2009). Reproductive assessment of bats. Ecological and behavioral methods for the study of bats.

[b27] Racey PA, Swift SM (1981). Variations in gestation length in a colony of pipistrelle bats (*Pipistrellus pipistrellus*) from year to year. J. Reprod. Fertil.

[b28] Rambaldini DA, Brigham RM (2008). Torpor use by free-ranging pallid bats (*Antrozous pallidus*) at the northern extent of their range. J. Mammal.

[b29] Schmid J, Speakman JR (2009). Torpor and energetic consequences in free-ranging grey mouse lemurs (*Microcebus murinus*): a comparison of dry and wet forests. Naturwissenschaften.

[b30] Speakman JR (2008). The physiological costs of reproduction in small mammals. Philos. Trans. R. Soc. Lond., B, Biol. Sci.

[b32] Speakman JR, Rowland A (1999). Preparing for inactivity: how insectivorous bats deposit a fat store for hibernation. Proc. Nutr. Soc.

[b33] Speakman JR, Thomas DW, Kunz TH, Fenton MB (2003). Physiological ecology and energetics of bats. Bat ecology.

[b34] Storey KB, Heldmaier G, Rider MH (2010). Mammalian hibernation: physiology, cell signaling, and gene controls on metabolic rate depression. Top. Curr. Genet.

[b35] Studier EH (1981). Energetic advantages of slight drops in body temperature in little brown bats, *Myotis lucifugus*. Comp. Biochem. Physiol.

[b36] Turbill C, Geiser F (2006). Thermal physiology of pregnant and lactating female and male long-eared bats, *Nyctophilus geoffroyi* and *N. gouldi*. J. Comp. Physiol. B.

[b37] Weather Underground Inc (2013). http://www.wunderground.com/history/.

[b38] Webb PI, Speakman JR, Racey PA (1993). The implications of small reductions in body temperature for radiant and convective heat loss in resting endothermic brown long-eared bats (*Plecotus auritus*. J. Therm. Biol.

[b39] Wilde CJ, Knight CH, Racey PR (1999). Influence of torpor on milk protein composition and secretion in lactating bats. J. Exp. Zool.

[b40] Willis CKR (2007). An energy-based body temperature threshold between torpor and normothermia for small mammals. Physiol. Biochem. Zool.

[b41] Willis CKR, Brigham RM (2007). Social thermoregulation exerts more influence than microclimate on forest roost preferences by a cavity dwelling bat. Behav. Ecol. Sociobiol.

[b42] Willis CKR, Brigham RM, Geiser F (2006). Deep, prolonged torpor by pregnant, free-ranging bats. Naturwissenschaften.

